# Quantitative GPCR and ion channel transcriptomics in primary alveolar macrophages and macrophage surrogates

**DOI:** 10.1186/1471-2172-13-57

**Published:** 2012-10-26

**Authors:** Paul J Groot-Kormelink, Lindsay Fawcett, Paul D Wright, Martin Gosling, Toby C Kent

**Affiliations:** 1 Respiratory Disease Area, Novartis Institutes for Biomedical Research, Horsham, RH12 5AB, UK

**Keywords:** COPD, Microfluidics, TaqMan, Arrays, High-throughput

## Abstract

**Background:**

Alveolar macrophages are one of the first lines of defence against invading pathogens and play a central role in modulating both the innate and acquired immune systems. By responding to endogenous stimuli within the lung, alveolar macrophages contribute towards the regulation of the local inflammatory microenvironment, the initiation of wound healing and the pathogenesis of viral and bacterial infections. Despite the availability of protocols for isolating primary alveolar macrophages from the lung these cells remain recalcitrant to expansion *in-vitro* and therefore surrogate cell types, such as monocyte derived macrophages and phorbol ester-differentiated cell lines (e.g. U937, THP-1, HL60) are frequently used to model macrophage function.

**Methods:**

The availability of high throughput gene expression technologies for accurate quantification of transcript levels enables the re-evaluation of these surrogate cell types for use as cellular models of the alveolar macrophage. Utilising high-throughput TaqMan arrays and focussing on dynamically regulated families of integral membrane proteins, we explore the similarities and differences in G-protein coupled receptor (GPCR) and ion channel expression in alveolar macrophages and their widely used surrogates.

**Results:**

The complete non-sensory GPCR and ion channel transcriptome is described for primary alveolar macrophages and macrophage surrogates. The expression of numerous GPCRs and ion channels whose expression were hitherto not described in human alveolar macrophages are compared across primary macrophages and commonly used macrophage cell models. Several membrane proteins known to have critical roles in regulating macrophage function, including CXCR6, CCR8 and TRPV4, were found to be highly expressed in macrophages but not expressed in PMA-differentiated surrogates.

**Conclusions:**

The data described in this report provides insight into the appropriate choice of cell models for investigating macrophage biology and highlights the importance of confirming experimental data in primary alveolar macrophages.

## Background

Macrophages play important roles in host defence, wound healing, immune regulation and muscle regeneration. In particular, the central role of the macrophage in removing necrotic cellular debris, shaping the inflammatory microenvironment and their key role in the pathogenesis of viral infection have led to an intense interest in furthering understanding of the alveolar macrophage within the field of respiratory research.

Chronic obstructive pulmonary disorder (COPD) is one of the most common respiratory diseases and represents a major public health problem. The World Health Organisation estimates that COPD was the fifth leading cause of death worldwide in 2002 and by 2030 will become the third leading cause of death. The disease is typically progressive and characterised by chronic airflow limitation caused by a mixture of small airway disease (obstructive bronchiolitis) and parenchymal destruction (emphysema). Key pathological changes in COPD include a marked increase in macrophages within the airways, lung parenchyma and pulmonary vasculature [1]. Macrophages are the predominant inflammatory cell within the lung where they secrete a wide range of inflammatory mediators and proteases. Combined with the phagocytic properties of a macrophage the large diversity of their biological functions can account for most of the known features of COPD [2]. Consequently, the *in-vitro* study of macrophage biology is a key activity within the COPD research area.

Macrophages have long been known to be produced by the differentiation of monocytes following their recruitment from blood-vessels into surrounding tissues [3]. Primary macrophages isolated from resident tissues cannot be readily expanded for prolonged culture, which limits their routine use for biological studies and drug discovery; therefore alternative cellular models more amenable to cell culture are frequently used as a surrogate for the investigation of macrophage biology. Commonly used macrophage surrogates include the circulating monocytic precursor cell, monocyte derived macrophages (MDMs), *in-vitro* differentiated promonocytic THP-1 and U937 cells or promyelocytic HL-60 leukaemia cells. It is unclear how well these surrogate systems recapitulate the biology of the primary macrophage for which they are intended to model; indeed, major phenotypic differences such as altered chemokine release and phagocytic behaviour have been shown to separate the primary macrophage and commonly used surrogate cell types [4]. Enhancing our understanding of these differences will help enable the choice of appropriate macrophage model systems for investigating macrophage biology, modelling disease pathology *in-vitro* and for establishing appropriate cell based screening assays for drug discovery.

G-protein coupled receptors (GPCRs) and ion channels are large families of cell surface proteins and together comprise the molecular target of approximately 40% of all FDA approved drugs [5]. Integral membrane proteins such as receptors and ion channels are key regulators of cellular function and unsurprisingly are dynamically regulated at the transcriptional level with differentiated cell types displaying marked differences in expression [6]. As is typical for membrane bound signalling proteins, the amount of GPCR and ion channel transcript is often low within the cell and at the limit of detection for DNA microarray technologies [7,8]. Quantitative PCR techniques such as TaqMan offer a higher sensitivity for detecting low-abundance transcript, but until recently have been limited to small scale gene expression studies due to their low throughput. Only recently with the advent of high density microfluid-based TaqMan arrays has the large scale quantitative analysis of whole, low-abundance gene families become feasible.

The aim of this study was to investigate how the expression of GPCR and ion channel genes differ between cell systems commonly used to model macrophage function. In this study we report for the first time a quantitative TaqMan-based transcriptional profile of entire families of integral membrane proteins, across several macrophage-like cell types, providing insights into their distinct gene signatures and identifying novel macrophage markers and potential future drug targets.

## Results

### Validation of TaqMan arrays for high-throughput quantitative gene expression

We validated the use of high-throughput 384-well TaqMan microfluidic cards for transcript expression studies by evaluating the inherent intraplate and interplate variability associated with these arrays. Identical copies of the same cDNA sample were profiled across two nuclear receptor 384-well arrays containing triplicate copies of gene specific primer and probe pairs. (*i.e*. six copies per unique primer/probe pair). When loading 100ng/port we found very high reproducibility within and between arrays and determined that a cut-off value of 32 C_T_ generates data with a >0.99 coefficient of determination both within and between arrays (Additional file 1: Figure S1).

To check for primer/probe efficiencies we analysed 4 ion channel arrays loaded with varying amounts of cDNA (from 1 to 1000ng/port). We investigated the correlation between template concentrations and C_T_ values and observed a linear decrease in C_T_ values with increasing template concentration thus demonstrating the quantitative validity of these HT-TaqMan arrays over a 3 log template concentration range (Additional file 1: Figure S2). However, at the lowest DNA concentration (1ng/port) very low expressing genes were not detected when applying a 32 C_T_ cut-off. Furthermore, at the highest concentration tested (1000ng/port) the highly expressed 18S housekeeping gene started to exhibit a loss of linearity indicating saturation of the primer and probes in the PCR reaction. To balance the requirements for sensitivity and the ability to quantify changes in highly expressed genes, 100ng of cDNA per port was used for all subsequent experiments.

### Comparison of global GPCR and ion channel expression in primary alveolar macrophages versus surrogate cell types

We examined the expression of 367 non-sensory GPCRs and 330 ion channels in primary human alveolar macrophages and five other cell types commonly used as *in-vitro* surrogates for primary macrophages; GM-CSF differentiated monocyte derived macrophages, isolated circulating monocytes and phorbol ester differentiated HL-60, U937 and THP-1 cells. As the expression of housekeeper genes can sometimes differ between cell types, the expression of GPCRs and ion channels were normalised against a large panel of housekeeping genes. The expression data for each individual gene was correlated against expression in primary alveolar macrophages and the data was fitted by linear regression.

**Figure 1:**
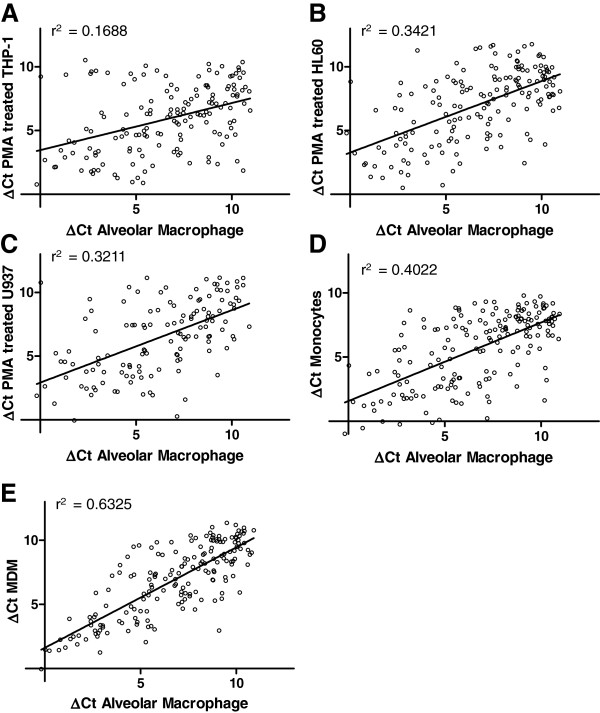
**Correlation of GPCR gene expression in alveolar macrophages in comparison to (A) PMA treated THP-1 cells (B) PMA treated HL60 cells (C) PMA treated U937 cells (D) peripheral blood monocytes and (E) monocyte derived macrophages.** Expression is reported as ΔC_T_ compared to the mean C_T_ of the housekeeping genes and each value is the mean of ≥3 biological replicates.

**Figure 2:**
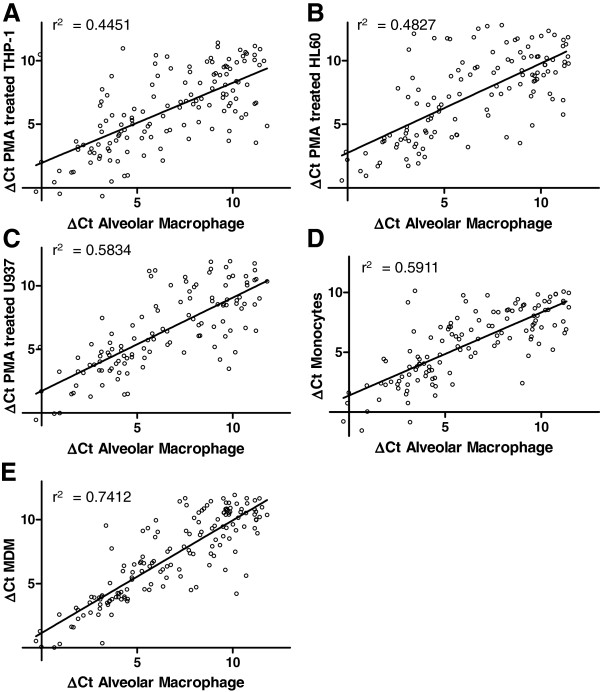
**Correlation of ion channel gene expression in alveolar macrophages in comparison to (A) PMA treated THP-1 cells (B) PMA treated HL60 cells (C) PMA treated U937 cells (D) peripheral blood monocytes and (E) monocyte derived macrophages.** Expression is reported as ΔC_T_ compared to the mean C_T_ of the housekeeping genes and each value is the mean of ≥3 biological replicates.

As demonstrated in Figures 1 and 2, the global GPCR and ion channel expression profiles differ markedly between the five cell types tested, with greatest correlation between MDMs and alveolar macrophages. The rank order of similarity for GPCRs was MDM (r^2^ = 0.6325) > monocyte (r^2^ = 0.4022) > HL60 (r^2^ = 0.3421) > U937 (r^2^ = 0.3211) > THP-1 (r^2^ = 0.1688) and for ion channels MDM (r^2^ = 0.7412) > monocyte (r^2^ = 0.5911) > U937 (r^2^ = 0.5834) > HL60 (r^2^ = 0.4827) > THP-1 (r^2^ = 0.4451). The expression profiles indicate that MDMs and monocytes have the most similar expression profiles compared with primary alveolar macrophages. Interestingly, the data indicate that the similarity in ion channel expression between alveolar macrophages and the surrogate cell types is greater than for GPCRs which may reflect greater dynamic transcriptional regulation of GPCRs compared with ion channels.

### Differential expression of individual GPCRs and ion channels in primary alveolar macrophages and surrogate cell types

**Figure 3:**
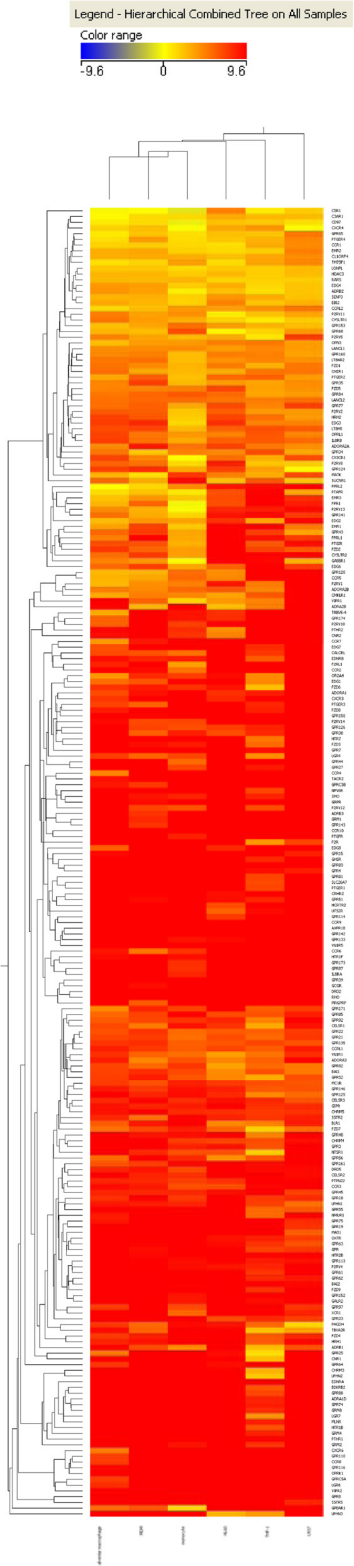
**Clustered heat map of ΔC**_**T**_**values from GPCR arrays.** Clustering of samples and of the genes to generate the heat maps was achieved using GeneSpring 11.5.1 software (Agilent Technologies Inc.). Unsupervised hierarchical clustering was carried out on genes using a Euclidean distance/similarity metric and a centroid linkage rule.

**Figure 4:**
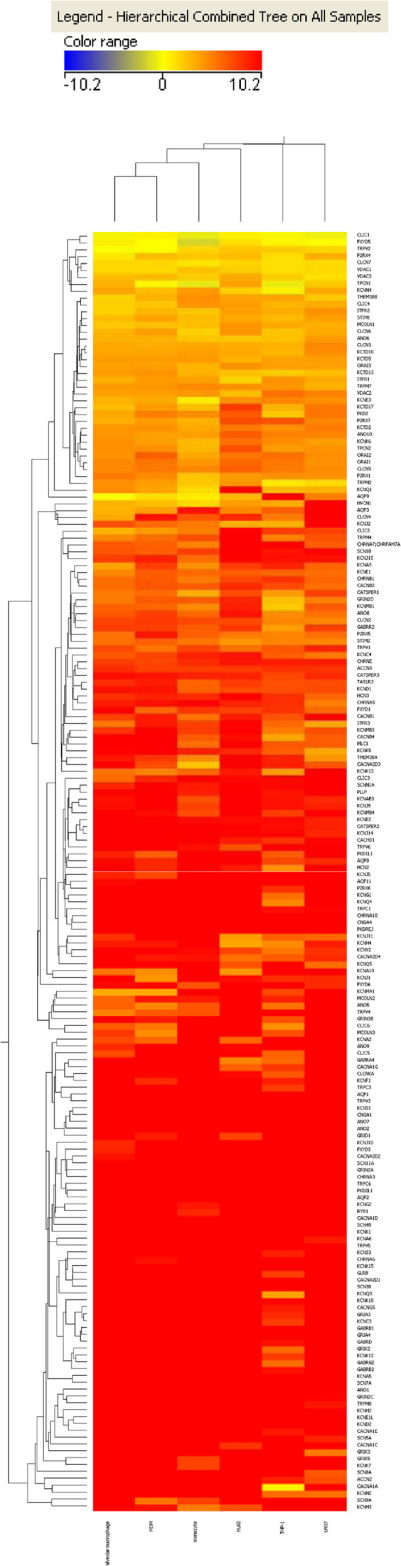
**Clustered heat map of ΔC**_**T**_**values from ion channel arrays.** Clustering of samples and of the genes to generate the heat maps was achieved using GeneSpring 11.5.1 software (Agilent Technologies Inc.). Unsupervised hierarchical clustering was carried out on genes using a Euclidean distance/similarity metric and a centroid linkage rule.

In order to identify individual genes differentially expressed between alveolar macrophages and the surrogate cell types we performed an unsupervised hierarchical clustering of genes using a Euclidean distance metric and a centroid linkage rule. In agreement with the correlation plots it was found that monocytes and MDMs have the most similar GPCR and ion channel expression profiles when compared with primary alveolar macrophages. Figures 3 and 4 show clustered heat maps of GPCR and ion channel expression across all cell types analysed. The overall number of GPCR and ion channel genes expressed is surprisingly high in all of the cell types with 164 non-sensory GPCRs and 115 ion channels expressed with mean C_T_ values <32 in alveolar macrophages and similar numbers expressed in all other cell types analysed.

The most highly expressed GPCR in the alveolar macrophages is the complement 5a receptor (C5R1) which is involved in regulating macrophage chemotaxis, activation and degranulation [9]. This gene was found to be expressed at similar levels in all surrogate cell types except the PMA-treated HL60 cell line where expression was 30-fold lower than the alveolar macrophages. The C3AR1 complement receptor was the third highest receptor in alveolar macrophages but expressed >13 fold lower in PMA-treated HL60s. Phorbol ester-differentiated HL60 cells have been reported to display many morphological and functional characteristics of primary macrophages, such as adherence to plastic and an increase in lysozyme and acid phosphatase synthesis, but demonstrate no zymosan-induced respiratory burst or phagocytosis of complement-opsonized *E.coli*[10]. The lack of such functional responses may be contributed to by a low level of complement receptor expression in HL60s in comparison with primary macrophages.

Formyl peptide like receptor 2 (FPRL2) is the second most highly expressed GPCR in the alveolar macrophages. Expression of FPRL2 was previously reported in MDMs and primary macrophage cell subtypes which are chronically exposed to pathogens, such as alveolar macrophages [11]. We found that only MDMs exhibit comparably high FPRL2 expression levels, with expression in monocytes and PMA differentiated cell lines being >20x and >400x lower, respectively.

Several other GPCRs that were highly expressed in alveolar macrophages were essentially absent in the surrogate models tested. Interestingly, several chemokine receptors were found to have substantially higher expression in alveolar macrophages compared to surrogates, particularly with respect to PMA differentiated cells. For example, the chemokine receptor CXCR6 was barely detectable in MDMs and monocytes (>1000× lower expression) and was not expressed in PMA differentiated surrogates. Similarly, the chemokine receptor CCR8 shows no detectable expression in the PMA differentiated cell lines and only very weak expression in a few individual monocyte and MDM replicates. Marked differences of expression were also observed for several other chemokine receptors including CCR4, CCR5, CCR6, CCR7, and CXCR3 (Additional file 2: Table S1). Other GPCRs which demonstrated dramatically reduced expression in the surrogate cell types compared to primary macrophages include the retinoic acid inducible orphan receptor GPRC5A and the adhesion receptors GPR110 and GPR116. The function of these orphans in AMs is not currently known.

The most highly expressed ion channel in the alveolar macrophages on the array is the chloride intracellular channel-1 (CLIC1). This channel was initially cloned from U937 cells and is expressed at very similar levels across all of the cell types. β-amyloid stimulation of microglial cells results in the production of TNF-α through activation of CLIC1 and it is interesting to speculate if CLIC-1 may play a similar stimulatory role in alveolar macrophages. The transient receptor potential vanilloid-4 channel (TRPV4) has been previously reported to be expressed in alveolar macrophages and has been suggested to play a central role in the initiation of ventilator-induced lung injury via the activation of AMs [12,13]. We also found that TRPV4 was highly expressed in AMs, MDMs and monocytes but surprisingly was not expressed in PMA-differentiated macrophage surrogates (Additional file 2: Table S2).

## Discussion

In this study the application of high throughput PCR has enabled the quantification of transcript expression of entire target families across multiple cell types. The GPCR and ion channel transcriptome of primary AMs was compared with several cell types commonly used to model macrophage function. Given the dynamic transcriptional control of GPCR and ion channel families and their important role in modulating cellular function, the changes in gene expression quantified in this study should provide a good measure of functional similarities or differences between different macrophage-like cell types [6,14].

The data in this study suggests that the widely used AM models are poor substitutes for primary AMs isolated from BAL. Surprisingly, we found that the GPCR and ion channel expression profile of commonly used PMA-differentiated promonocytic and promyelocytic surrogates was less primary AM-like than non-treated peripheral blood monocytes. GM-CSF differentiated monocyte-derived macrophages appear to be most similar to AMs with respect to GPCR and ion channel expression but nonetheless demonstrate many significant differences in the expression of genes known to play critical roles in regulating AM function.

It is over 50 years since the first report described the isolation of macrophages from BAL fluid, yet no method has since been established for the culturing and expansion of these cells [15]. Consequently, surrogate cell types continue to be widely employed to interrogate the biology of the macrophage and to establish high throughput screening assays for drug discovery. An improved understanding of the GPCR and ion channel expression profiles of AMs and AM surrogates will provide valuable information on the design and interpretation of experiments utilising macrophage surrogates.

Exacerbations remain a major cause of hospitalization, mortality, and morbidity in COPD patients, with viral or bacterial infections representing a principle aetiological factor [16]. Alveolar macrophage dysfunction in COPD patients is thought to contribute to an increased susceptibility to exacerbations through impaired phagocytosis, chemotaxis, and cytokine production leading to an impaired host defence [2,17,18]. Many cell surface receptors including GPCRs and ion channels participate in the regulation of this process and may thus play in important role in regulating host immunity [9,19,20]. The elucidation of differential GPCR and ion channel expression in primary AMs versus macrophage surrogates will help to refine the choice of appropriate models for investigating macrophage biology. For example, the high-affinity CCL1 receptor, CCR8, has previously been shown to be highly expressed on AMs and its expression has been associated with phagocytic macrophages [21]. Furthermore, CCR8 is upregulated on AMs from COPD patients and is reported to impact macrophage adhesion, chemotaxis, ROS generation and cytokine production [22]. In this study we report that although CCR8 is highly expressed on primary AMs, it is essentially absent on all evaluated macrophage surrogates.

Macrophages play a crucial role in the pathogenesis of viral infections, are often among the first cells to be infected and can act as a reservoir for further spread to other cell types [23,24]. Numerous chemokine GPCRs have been previously shown to be expressed in AMs [25], furthermore, many viruses utilise chemokine GPCR co-receptors to mediate membrane fusion and promote viral entry into target cells; for example CCR5 and CXCR4 are the principle HIV-1 co-receptors mediating T cell and macrophage tropism with T-cell tropic viruses using CXCR4 [26], whereas macrophage tropic HIV-1 viruses primarily utilise CCR5 as a co-receptor [27]. In this study we found that CCR5 and several other chemokine receptors which support viral infection, such as CCR8 and CXCR6 are either absent or demonstrate markedly different expression levels in macrophage-like cell lines compared with primary AMs. Both CCR8 and CXCR6 are highly expressed in primary AMs but are not expressed in PMA-differentiated surrogates and are expressed at below levels of quantification in both MDMs and primary monocytes. CCR5 is highly expressed in AMs but has >200 fold lower expression in PMA-differentiated THP-1 and U937 cells. These findings are of particular interest as macrophage surrogates are widely used to model changes in cellular tropism of viral variants [28], however in contrast to primary isolated macrophages viral isolates often lack the capacity to infect and replicate in phorbol ester-differentiated promonocytic and promyelocytic cells [29,30]. The importance of chemokine receptor signalling in regulating macrophage function and the stark differences in chemokine expression from cell types described in this study highlights the importance of using primary cells to investigate macrophage biology wherever possible. A better understanding of the GPCR and ion channel expression profiles of primary AMs and surrogate cell models will likely inform the use of appropriate macrophage models for studying different aspects of macrophage biology. The application of high-throughput TaqMan arrays to better understand changes in gene expression in diseased tissues is also likely to further our understanding of disease aetiology and progression and may lead to the identification of novel drug targets.

Several different protocols may be used to drive promonocytic or promyelocytic cell lines into macrophage-like cells in order to generate model systems for studying macrophage biology and the protocols used can impact the resulting cellular phenotype [4,31]. Furthermore, it is well established that primary macrophages display marked phenotypic heterogeneity covering a spectrum of classically and alternatively activated phenotypes [32]. It should also be remembered that this study has only compared GPCR and ion channel expression and not the entire transcriptome. Furthermore, macrophages from different tissues and even within the same tissue can show considerable phenotypic heterogeneity [33]. Additionally, it is unclear to what extent primary AMs isolated from BAL fluid are representative of the AM population resident within the alveoli [34]. Further studies will be required to better understand what impact the different protocols used to establish macrophage surrogates may have on the resulting gene expression profiles and perhaps better understand how the resulting surrogates reflect the many primary macrophage phenotypes observed within physiological and pathophysiological settings.

## Conclusions

Macrophages play an important role in regulating many biological processes including host defence and muscle regeneration. In this study we have validated the use of high-throughput microfluid-based TaqMan arrays for detecting quantitative changes in gene expression profiles and applied this technology to the quantitative analysis of macrophage gene expression. To our knowledge this is the first study to describe the expression of the entire non-sensory GPCR and ion channel families in AMs and macrophage surrogates. We have identified multiple genes whose expression were hitherto not described in human alveolar macrophages and provide a platform for future studies to further explore the impact that GPCRs and ion channels may have on the macrophage phenotype. These data may also provide researchers with novel macrophage markers and potential future drug targets.

Without access to a plentiful supply of primary macrophages, surrogates continue to be widely used to model macrophage function and will likely continue to form a cornerstone of the *in-vitro* methodologies employed to investigate macrophage biology. This study provides insight into the observed phenotypic characteristics of surrogates and informs on the use of most appropriate models. However, the differences observed in GPCR and ion channel expression described in this study highlights the importance, wherever possible, of using primary cells for the *in-vitro* modelling of alveolar macrophage behaviour.

## Methods

### Preparation of cDNA samples from human donors and cell lines

Monocytes were isolated from blood from 5 healthy human donors using the Monocyte Isolation kit II (Miltenyi Biotec, Surry, UK) according to the manufacturer's instructions. Monocyte derived macrophages (MDMs) were generated by treating freshly isolated monocytes with 5 ng/ml GM-CSF (R&D systems, Abingdon, UK) for 10 days in culture. Alveolar macrophages were isolated from bronchoalveolar lavage (BAL) fluid from an additional 5 healthy, non-smoker, donors as previously described [35]. HL60, U937 and THP-1 cell lines were obtained from ECACC (Salisbury, UK). HL60 cells were cultured in Iscove's modified Dulbecco's medium with 2 mM L-glutamine and 20% FCS. U937 and THP-1 cells were cultured in RPMI 1640 medium with 2mM L-glutamine and 10% FCS (all media components from Invitrogen, Paisley, UK). For PMA treatment cells were cultured for 48 hrs in media containing 16ng/ml PMA (Sigma Aldrich, Poole, UK).

For all samples RNA was isolated from 5 million cells using the RNAqueous kit (Invitrogen, Paisley, UK) and subsequently treated with TURBO DNase (Invitrogen, Paisley, UK) to remove genomic DNA. cDNA was synthesized using a High Capacity RNA-to-cDNA kit (Invitrogen, Paisley, UK) according to manufacturer's instructions.

### HT-TaqMan arrays

A bespoke 384-well ion channel HT-TaqMan array was designed containing validated primer/probe sets for the complete ion channel gene family (330 genes – several genes having multiple primer/probe sets) and primer/probe sets for 11 house keeping genes (ACTB, ACTN1, B2M, GAPDH, GUSB, HPRT, PGK1, PPIA, RPLO, TBP, and TFRC) for the purpose of quality control and data normalisation (Invitrogen, Paisley, UK). GPCR arrays contained validated primer/probe sets for 367 GPCRs and primer/probe sets for 13 house keeping genes (ACTB, B2M, GAPDH, GUSB, HMBS, HPRT, IPO8, PGK1, POLR2A, PPIA, RPLO, TBP, and TFRC) (Invitrogen, Paisley, UK). Nuclear receptor arrays contained 48 nuclear receptor genes replicated 6 times per 384-well array (Invitrogen, Paisley, UK). A full list of genes for the ion channel and GPCR arrays with their primer/probe identifiers can be found in the supplementary methods section.

HT-TaqMan arrays were run on an Applied Biosystems 7900HT fast real-time PCR instrument according to the manufacturer's instruction. Unless otherwise stated, 100ng of cDNA was loaded per port on each array. Data was analysed using RQ manager version 1.2 and DataAssist version 2 (Applied Biosystems) with a C_T_ threshold set at 0.2 for all samples. All housekeeping genes were selected for normalisation of the data.

For the heat map analysis the data was analysed in DataAssist with a maximum allowable C_T_ value of 40. Only genes with a mean C_T_ values ≤32 for at least one cell type were included in the analysis. Δ C_T_ values were calculated relative to all housekeeping genes present on each array, as described above. Clustering of samples and of the genes to generate heat maps was achieved using GeneSpring 11.5.1 software (Agilent Technologies Inc.). Unsupervised hierarchical clustering was carried out on genes using a Euclidean distance/similarity metric and a centroid linkage rule.

For the scatter plot analysis the data was analysed in DataAssist with a maximum allowable C_T_ value of 32. Δ C_T_ values were calculated relative to all previously described housekeeping genes. Mean Δ C_T_ values were calculated for each gene in each sample group and the data was plotted as scatter plots for each cell type in comparison to primary alveolar macrophages in GraphPad Prism 5 (GraphPad Software Inc.). Data was fitted to a linear regression to calculate coefficient of variation (r^2^) values.

## Abbreviations

AM: Alveolar macrophage; BAL: Bronchoalveolar lavage; COPD: Chronic obstructive pulmonary disorder; GM-CSF: Granulocyte-macrophage colony-stimulating factor; GPCR: G-protein coupled receptor; MDM: Monocyte-derived macrophage; PCR: Polymerase chain reaction; PMA: phorbol-12-myristate-13-acetate.

## Competing interests

The authors declare that they have no competing interests.

## Authors' contributions

LF, PGK, and PW carried out the TaqMan array studies. MG, PGK and TK conceived of and participated in the design of the study. PGK, LF and TK performed the statistical analysis and drafted the manuscript. All authors read and approved the final manuscript.

## Supplementary Material

Additional file 1 **Figure S1.** TaqMan array intraplate and interplate variability. **Figure S2.** Primer/probe efficiencies. Click here for file

Additional file 2 **Table S1.** GPCR raw data. **Table S2.** Ion Channels raw data. Click here for file
